# Surgical Excision with Adjuvant Therapies in the Management of Keloids: A Systematic Review

**DOI:** 10.3390/medicina62050916

**Published:** 2026-05-08

**Authors:** Monika Wojarska, Klaudia Kokot, Brygida Ossowska, Wiktoria Borzyszkowska, Hanna Szóstek, Amelia Stolp, Izabela Zakrzewska, Zuzanna Durska, Julia Wojciechowska, Bogdan Jabłoński, Jerzy Jankau

**Affiliations:** 1 Plastic Surgery Clinic, Medical University of Gdańsk, Smoluchowskiego, 80-214 Gdansk, Poland; jerzy.jankau@gumed.edu.pl; 2 Students’ Scientific Circle of Plastic Surgery, Department of Plastic Surgery, Medical University of Gdańsk, Smoluchowskiego, 80-214 Gdansk, Poland; brygida.ossowska@gumed.edu.pl (B.O.); wborzyszkowska@gumed.edu.pl (W.B.); hannaszostek@gumed.edu.pl (H.S.); ameliastolp@gumed.edu.pl (A.S.); izabelazakrzewska@gumed.edu.pl (I.Z.); zuzanna.durska@gumed.edu.pl (Z.D.); bogdan.jablonski@gumed.edu.pl (B.J.); 3 Scientific Circle of Neurotraumatology, Department of Emergency Medicine, Medical University of Gdansk, Smoluchowskiego, 80-214 Gdansk, Poland

**Keywords:** keloid treatment, keloid surgical treatment, adjuvant therapies, systematic review

## Abstract

*Background and Objectives*: Keloids are fibroproliferative disorders marked by excessive fibroblast activity, abnormal collagen deposition, and impaired wound healing. They are frequently associated with pain, pruritus, and significant aesthetic concerns, leading to reduced quality of life. Surgical excision alone is burdened by high recurrence rates, underscoring the need for effective adjuvant therapies. This systematic review aimed to assess the effectiveness of surgical excision combined with adjuvant physical and pharmacological therapies in keloid management, with particular emphasis on recurrence rates. *Materials and Methods:* The review was conducted in accordance with PRISMA guidelines. A systematic search of PubMed and Web of Science identified studies evaluating surgical excision of keloids with adjunctive therapies. Twenty-one studies involving more than 8627 patients met the inclusion criteria. Extracted data included study design, patient and lesion characteristics, treatment modalities, and recurrence rates. Due to marked heterogeneity among treatment protocols, a meta-analysis was not performed. *Results:* Among physical adjuvant therapies, postoperative brachytherapy showed the lowest recurrence rates (3.1–15%), outperforming radiotherapy and external-beam radiation therapy (14–29.3%). Compression therapy achieved recurrence rates of 10.66% and 14%, particularly effective in auricular keloids. Pharmacological adjuvant therapies demonstrated variable efficacy. Triamcinolone acetonide injections were associated with recurrence rates ranging from 6.6% to 33%, depending on the protocol. Adjuvant 5-fluorouracil reduced recurrence compared with surgery alone, whereas imiquimod 5% showed higher and less consistent recurrence rates. Combination pharmacological therapies consistently yielded better outcomes than monotherapy. *Conclusions:* Surgical excision combined with adjuvant therapy is the most effective strategy for keloid treatment. Multimodal approaches significantly reduce recurrence compared with surgery alone. However, substantial heterogeneity in lesion characteristics, treatment timing, and therapeutic protocols limits comparability between studies. Further high-quality, standardised clinical trials are needed to optimise management strategies and develop evidence-based guidelines.

## 1. Introduction

A keloid is a fibroproliferative disorder characterized by excessive fibroblast activation and dysregulated wound healing [1]. It is associated with abnormal collagen deposition and sustained inflammatory activity [2]. These pathological processes are typically triggered by cutaneous injury or surgical procedures [1]. Unlike hypertrophic scars, which stay limited to the primary wound margins and may partially regress over time, keloids extend beyond the boundaries of the initial injury and do not undergo spontaneous regression [3]. Histologically, hypertrophic scars are characterized by predominantly aligned type III collagen fibers, whereas keloid tissue contains disorganized bundles of both type I and type III collagen [4]. Clinically, keloids frequently cause pruritus, pain, and discoloration, leading to a substantial reduction in quality of life [5].

Although the exact pathogenesis remains incompletely understood, it is considered multifactorial, involving dysregulated immune responses and impaired wound healing mechanisms [5]. A genetic predisposition has also been identified, with a higher incidence reported among individuals of African and Asian origin [5], as well as in anatomical regions exposed to increased mechanical stress, including the chest, shoulders, upper back, and earlobes [6].

Over the past decades, numerous therapeutic approaches have been proposed for the management of keloids; however, no single modality has demonstrated universal effectiveness.

Commonly applied interventions for keloid management include physiotherapy, pharmacological treatments, surgical excision, and biological therapies [7]. To enhance treatment efficacy and reduce recurrence, multimodal strategies combining approaches from different therapeutic categories are increasingly adopted. Treatment selection is influenced by lesion size, anatomical location, and individual patient response. Given the pathophysiological mechanisms underlying keloid formation and the high recurrence rates associated with surgical excision alone, surgery should be incorporated into combined therapeutic regimens rather than used as a standalone intervention [7].

The objective of this review is to synthesize current scientific evidence on keloid management, with particular emphasis on the efficacy of surgical excision combined with adjunctive therapies. By systematically analysing clinical outcomes, recurrence rates, and the methodological rigor of existing studies, this review aims to define the current state of knowledge and identify key gaps requiring further investigation. Despite substantial progress the development of an optimal, evidence-based, and universally applicable treatment algorithm for keloids remains an ongoing challenge.

## 2. Materials and Methods

This study adhered to the Preferred Reporting Items for Systematic Reviews and MetaAnalyses (PRISMA) guidelines [8] (The PRISMA 2020 Checklist is provided in the Supplementary Materials). A systematic literature search was conducted in the PubMed and Web of Science databases to identify studies published up to 2005 that evaluated surgical excision of keloids combined with complementary treatment modalities: physical methods (brachytherapy, radiotherapy, external-beam radiation therapy, compression therapy) and pharmacological methods (5-Fluorouracil, triamcinolone, imiquimod 5% and combination therapies). The protocol was prospectively registered in the PROSPERO database (registration ID:CRD420261335714).

The following search strategy was used:

(“keloid treatment” OR “keloid surgical treatment”) AND (“systematic review” OR “metaanalysis” OR “metaanalysis”).

Unpublished studies and articles written in languages other than English were excluded. During the initial screening of titles and abstracts, abstracts, case reports, conference proceedings, letters, and editorials were excluded. Duplicate records identified across databases were removed using Mendeley software (Reference Manager, version 2.130.2).

The initial database search was independently performed by three researchers. After duplicate removal, two reviewers independently screened the remaining titles and abstracts according to predefined inclusion and exclusion criteria. Full-text articles deemed potentially relevant were subsequently retrieved and assessed for eligibility. Only full-text studies reporting on the effectiveness of surgical excision supported by adjunctive therapies were included in the final review.

Any disagreements regarding study inclusion were resolved through discussion with the lead authors (M.W. and K.K.), with final decisions made under the supervision of the first author.

**Figure 1:**
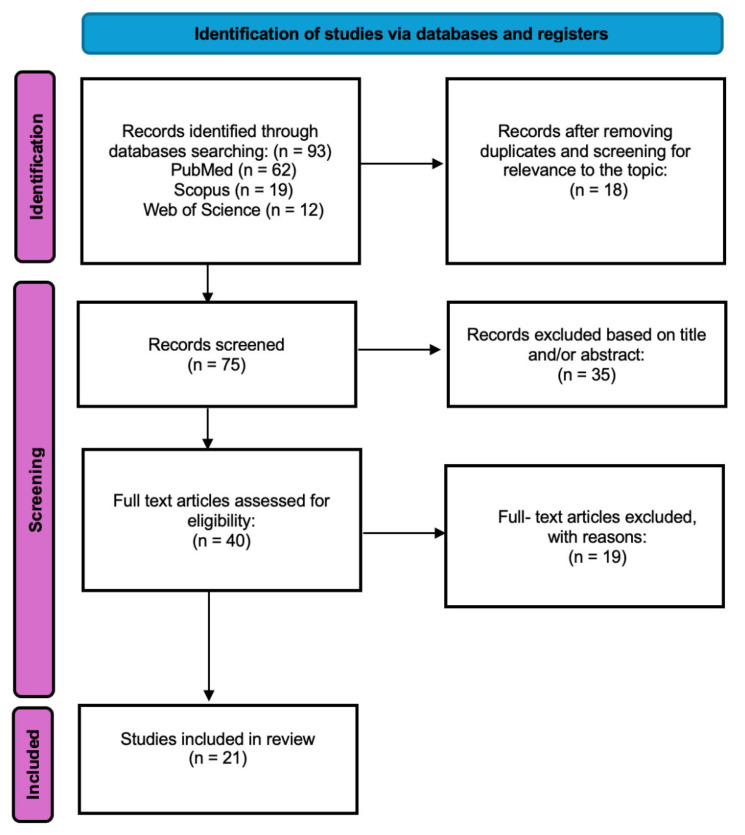
Prisma flowchart.

The study selection process is summarized in a PRISMA flow diagram (Figure 1).

The initial search identified 93 articles. After excluding articles that do not match the criteria 75 studies were included for abstract review. Finally, 40 were selected for full text appraisal of which 21 met all the inclusion criteria and were included in this review. This systematic review included 21 articles including over 8627 patients. The complete dataset is summarized in Table 1 and Table 2 and Supplementary Table S1.

Due to substantial heterogeneity in keloid treatment modalities, study designs, and outcome measures, the available literature was considered too diverse to permit a formal meta-analysis.

Following data was abstracted from original studies:

1. General study information: authors, publication year, country, institution.
2. Patient characteristics: number of patients, sex, age.
3. Recurrence rate.
4. Lesion localisation.

## 3. Results

### 3.1. Physical Methods

#### 3.1.1. Brachytherapy

In 4 studies describing a total of 3927 patients, brachytherapy was presented as an adjuvant method in the postoperative treatment of keloids. This modality was most frequently applied within 24 h following surgical excision. One study demonstrated a particularly low recurrence rate of 3.1% among 43 treated patients [9] whereas the remaining publications reported recurrence rates ranging from 9.7% to 15%, indicating generally favourable outcomes with this combined approach [10,11,12]. The timing of brachytherapy was consistent across studies, with all reporting initiation within 24 h postoperatively.

#### 3.1.2. Radiotherapy

In 10 of the analysed studies including a total of 2941 patients, radiotherapy was used as an adjuvant treatment modality, most administered within 72 h following surgical excision. In two studies, radiotherapy was initiated on the fourth postoperative day [13,14]. Another report, a triple-therapy approach combining surgical excision and radiotherapy with an additional modality (e.g., hyperbaric oxygen therapy, pressure therapy, platelet-rich plasma, or 5fluorouracil) resulted in a recurrence rate of 11.2% [15]. A study focusing on auricular keloids treated with adjuvant radiotherapy reported a recurrence rate of 4.8%, while a separate investigation using a combination of surgery, platelet-rich plasma, and superficial photon radiation achieved a 95.5% non-recurrence rate [16]. Across the remaining studies, reported recurrence rates ranged from 14% to 29.3% [13,14,17,18,19,20,21,22]. Overall, the timing of radiotherapy varied across studies, although most protocols involved initiation within the first 24–72 h after surgery.

#### 3.1.3. External-Beam Radiation Therapy (EBRT)

External-beam radiation therapy (EBRT) for the treatment of keloids was reported in three studies encompassing a total of 3130 patients. In one study, EBRT administered within 72 h after surgical excision resulted in recurrence rates of 16% for keloids located at nonauricular sites and 11% for auricular lesions [22]. In the remaining studies, reported recurrence rates ranged from 17% to 28.4%, with EBRT most applied within 24 h postoperatively [11,12]. The timing of EBRT was relatively consistent, with most studies reporting administration within 24–72 h postoperatively.

#### 3.1.4. Compression Therapy

Compression therapy was evaluated by the authors in 2 studies with reported recurrence rates were 10.66% and 14%, suggesting that this non-invasive modality may provide satisfactory outcomes in reducing keloid recurrence. However, the effectiveness varied depending on treatment protocols and anatomical location [11,23]. The timing of compression therapy was not clearly defined and was inconsistently reported across studies.

### 3.2. Pharmacological Methods

Data from studies investigating the use of 5-fluorouracil (5-FU), triamcinolone (TAC), and Imiquimod 5% as adjuvant therapies following surgical treatment of keloids were analysed.

#### 3.2.1. 5-Fluorouracil (5-FU)

The analysis included 3 studies, encompassing a total of 282 patients who received 5-FU as an adjuvant treatment administered immediately following surgical excision.

While no recurrences were reported in the included studies, one study [24] described a reduction in recurrence rates from 87% to 32% compared to surgical excision alone. In most studies, 5-FU was initiated immediately after surgery and continued as part of repeated injection protocols at defined intervals.

#### 3.2.2. Triamcinolone (TAC)

The use of TAC injections as a standalone surgical adjuvant was reported in 7 studies, involving a total of 1026 patients. Treatment protocols varied, most commonly administered within 7 to 30 days after surgery [25,26]. Two studies employed repeated injections at intervals ranging from 14 to 90 days [20,24], while two others used intraoperative TAC injections [11,27]. Reported recurrence rates varied considerably, with the lowest rate of 6.6% observed in the study by Zhang et al. (2024) and the highest rate of 33% reported by Reid et al. (2025) [9,25]. Overall, the timing of TAC administration was highly variable, ranging from intraoperative use to delayed postoperative regimens extending up to several weeks.

#### 3.2.3. Imiquimod 5%

The analysis was based on a single study that met the inclusion criteria [28]. The group consisted of 77 patients over the age of 12. The topical treatment was initiated either immediately after surgery or with a delay of up to seven days.

The meta-analysis revealed a relatively high, yet highly variable, recurrence rate of 39%. The timing of imiquimod initiation showed limited variability but was based on data from a single study.

#### 3.2.4. Combination Therapies

Studies evaluating combination pharmacological therapies were also analysed. In one study Bijlard et al., a dual regimen combining 5-FU and triamcinolone acetonide (TAC) was administered in a cohort of 24 patients on days 0, 28, and 56 [24]. The results demonstrated superior outcomes with combination therapy compared with 5-FU monotherapy. In another study involving 24 patients [29], a treatment protocol combining corticosteroid injections with self-administered topical steroid ointment was evaluated. The protocol included a single injection at the time of suture removal, followed by injections every two weeks for a total of five sessions; each injection consisted of 1 mL triamcinolone and 1 mL procaine hydrochloride. This approach resulted in a reported recurrence rate of 14.3%. The timing of combination therapies was heterogeneous, reflecting differences in individual components and treatment protocols.

*Follow-Up*

**Figure 2:**
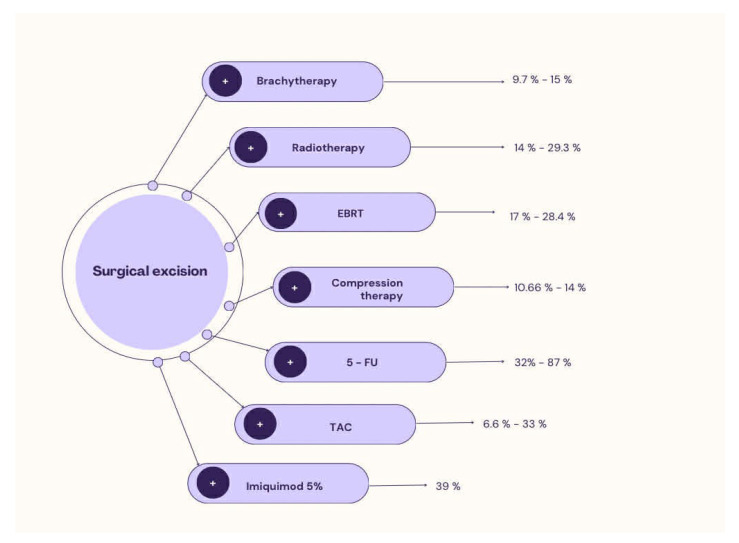
Recurrence rate of keloids after dual therapy flowchart.

The outcome assessment time was reported in 17 out of 21 studies; it was not standardized, and its range varied from 3–6 months up to 16 years. In only 2 studies was the follow-up period shorter than 12 months. The available data demonstrate heterogeneity both in the duration of follow-up and in the methods of its reporting. All the articles included in our study are presented in Table 1 and Table 2. Recurrence rate of keloids after dual therapy flowchart is presented in Figure 2.

**Table 1:** Overview of Studies Evaluating Adjunctive Methods in Surgery and Their Impact on Recurrence Rates.

Title	Author et al.	Year of Publication	Type of Study	How Many Works are Included	Number of Patients	Age (Years)	Body Area	Adjunctive Method in Surgery	Recurrence Rate Compared with Method Alone (%)	Recurrence Rate Compared to Surgery Alone (%)	Comparison of Recurrence Rates Between Surgery Alone and Adjunctive Method
Keloids and hypertrophic scars in individuals with darker Fitzpatrick skin types: a systematic review of treatment efficacy and quality of life outcomes	D. Reid [9]	2025	systematic review	14	776	no information	face, ears, neck, and other mobile/visible regions	brachytherapy + radiation therapy + intralesional triamcinolone (postoperative or intraoperative) + 5-fluorouracil injections	no information	surgery alone: recurrence rates up to 83% in some cases surgery + brachytherapy: 3.1% recurrence surgery + PRP + superficial photon radiation: 95.5% non-recurrence surgery + intraoperative triamcinolone: 0% recurrence after 12 months surgery + postoperative triamcinolone: 33% recurrence vs. 54% with surgery alone	Combined therapies yield better outcomes than surgery alone Improvement in scar size, symptoms (pain, pruritus), and quality of life
Post-keloidectomy irradiation using high-dose-rate superficial brachytherapy	Shigehiko Kuribayash [10]	2011	systematic review	no information	21	18–69	anterior chest wall, scapular region, lower jaw, suprapubic region, and other areas	brachytherapy	no information	no information	
What Do We Know About Treating Recalcitrant Auricular Keloids? A Systematic Review and Meta-Analysis	Luke R. R. Zawadiuk [11]	2022	systematic review and metaanalysis	13	no information	no information	auricular keloids	brachytherapy + compression therapy + external beam radiation therapy + intralesional steroid injection		no information	no information
Surgical Excision with Adjuvant Irradiation for Treatment of Keloid Scars: A Systematic Review	Michiel CE van Leeuwen [12]	2015	systematic review	33	3103	2–82	earlobe, chest, shoulder, neck, jawline	surgery + HDR brachytherapy LDR brachytherapy external radiation therapy	0–50%	no inf	no data
Combined surgical excision and radiation therapy for keloid treatment	Sadanori Akita [13]	2007	systematic review	no information	32	11–79 years old	anterior chest wall, scapular and back, abdomen and suprapubic, ear, neck, upper limb and lip	radiotherapy	no information	no information	Better choice is to combine surgery with radiotherapy.
Postoperative radiation protocol for keloids and hypertrophic scars: statistical analysis of 370 sites followed for over 18 months	Rei Ogawa [14]	2007	statistical analysis	no information	218	no information	auricle, earlobe, anterior chest wall, scapular region, suprapubic region, and other (upper limb, lower limb, and back)	radiotherapy	no information	no information	Better choice is to combine surgery with radiotherapy.
					109	no information	anterior chest wall, scapular region, and suprapubic region	radiotherapy	no information	no information	Better choice is to combine surgery with radiotherapy.
Wound Coverage, Adjuvant Treatments, and Surgical Outcomes for Major Keloid Scars: A Systematic Review and Meta-Analysis	David Cardenas [15]	2024	systematic review and metaanalysis	10	244	22–79	chest, face, neck, abdomen	surgery + post-excisional radiotherapy, triamcynolon acetonid,	much higher (lack of detail information).	patients receiving local flap coverage plus adjuvant radiotherapy were found to have significantly lower keloid recurrence rates and higher patient satisfaction	In essence, the evidence suggests that combining surgical excision with wound coverage (such as local flap reconstruction) and adjuvant radiotherapy yields superior outcomes—namely lower recurrence rates and higher patient satisfaction—when compared to surgery alone or with secondary-intention healing. The authors conclude that “surgical excision and radiation therapy [remain] the mainstay treatment for major keloids”.
Analysis of the surgical treatments of 63 keloids on the cartilaginous part of the auricle: effectiveness of the core excision method	Rei Ogawa [16]	2015	retrospective cohort study	no information	57	no information	earlobe	radiotherapy	no information	no information	Lower recurrence rates were achieved with surgery combined with radiotherapy.
Optimizing Radiotherapy for Keloids: A Meta-Analysis Systematic Review Comparing Recurrence Rates Between Different Radiation Modalities	Peter Mankowski [17]	2017	metaanalysis	72	no information but of the 98 treatment groups, 71 groups were treated by surgical excision first followed by radiotherapy (postexcisional radiotherapy)	no information	chest and trunk, upper extremity, lower extremity, head, and neck or ears	radiotherapy	radiotherapy alone 37%, radiotherapy + surgery 22%	no information	Lower recurrence rates were achieved with surgery combined with radiotherapy. The lowest recurrence rate of 15% was found for brachytherapy with 23% for electron beam and 23% for X-ray therapies.
Chest keloids: effect of surgical excision and adjuvant radiotherapy on recurrence, a systematic review and meta-analysis	Oliver J. Miles [18]	2021	systematic review and metaanalysis	12	400	no information	chest	surgery + post-excisional radiotherapy	post-excisional radiotherapy has recurrence of 22% compared to 37–43% for monotherapy	surgical excision alone has a recurrence rate of more than 50%, and up to 100% vs surgical excision + radiotherapy 22%	Recurrence 22% with surgery + radiotherapy; authors note improved outcomes versus surgery alone, though no standalone surgery data available.
Effectiveness of Core Excision Technique for Keloids: A Systematic Review	Xiaoye Ran [19]	2024	systematic review	20	926 keloids (no information about the patients)	9–61	ear other areas: face, trunk, limbs, perineum	surgical keloid core excision technique + radiotherapy surgical keloid core excision technique + steroid injections	the recurrence rates of keloids treated with adjuvant radiotherapy ranged from 0% to 14.1%, steroid injections 0% to 18.8%.	in the study that did not administer adjuvant therapy, the recurrence rate was 12.5% // previous studies have demonstrated that keloid scars treated solely with surgery exhibit a high recurrence rate, ranging from 45% to 100%	Previous studies have demonstrated that keloid scars treated solely with surgery exhibit a high recurrence rate, ranging from 45% to 100%. The recurrence rate falls below 50% when surgery is combined with intradermal corticosteroids, whereas it is less than 10% when external radiation therapy is applied after excision, often combined with other therapies. It is generally accepted that surgically treated keloids should undergo adjuvant therapy to reduce recurrence rates. Overall, core excision with adjuvant therapy effectively treats keloids, particularly auricular keloids, with a low recurrence rate. This technique represents a comparatively acceptable surgical method for treating keloids with a low recurrence rate when combined with adjuvant therapies. It can lead to good morphologic effects, especially for ear keloids, and is particularly beneficial for reconstructing large defects with low tension. Postexcision radiation therapy and intralesional steroid injection were the 2 most commonly used adjuvant therapy methods.
A Comparison of the Effectiveness of Triamcinolone and Radiation Therapy for Ear Keloids after Surgical Excision: A Systematic Review and Meta-Analysis	Jin Yong Shin [20]	2016	systematic review and metaanalysis	25	1105	no information	earlobe	triamcynolon, radiotherapy	authors note that surgery alone carries a much higher risk of recurrence, but no separate meta-analysis was performed for this group.	relapse rate (surgery + TAC) 15.4% relapse rate (surgery + radiotherapy) 14.0%	much better surgery + TAC or RT
The Efficacy of Surgical Excision Plus Adjuvant Multimodal Therapies in the Treatment of Keloids: A Systematic Review and Meta-Analysis	Morgan M Ellis [21]	2020	metaanalysis and systematic review	60	5547 keloids (no information about the patients)	no information	facial and non-facial	dual therapy: surgery + radiotherapy, surgery + TAC triple therapy: surgery + radio + 3rd, surgery + TAC + 3rd quadruple therapy: surgery + 3 others	no information	65–99%	Dual and triple therapy show a significantly lower relapse rate than monotherapy. The most effective method is triple therapy: surgical excision, radiation and 3rd: HBO or PRP recurrence rate is 7.7%.
Effect of the biologically effective dose of electron beam radiation therapy on recurrence rate after keloid excision: A meta-analysis	Na-Hyun Hwang [22]	2022	metaanalysis	28	3128 keloids (no information about the patients)	no information	whole body, ear	adjuvant electron beam radiation therapy after keloid excision	no information	the recurrence rate for all sites was 16% and for the ear 11%	Among the various treatments for keloids, postoperative radiotherapy is highly effective in reducing local recurrence. A higher biologically effective radiation dose (BED) is associated with a lower recurrence rate. Ear keloids respond more favorably than other locations.
Compression Therapy for Keloid Scars: A Systematic Review and Meta-analysis	Sadia M Tahir [23]	2024	systematic review	27	2281 keloids (no information about the patients)	10–30	auricular, head, chest, neck	Surgery + pressure earing, surgery + silicone gel	no information	no information	better outcomes with surgery + compression + silicone gel
Intralesional 5-fluorouracil in keloid treatment: a systematic review	Eveline Bijlard [24]	2015	metaanalysis and systematic review	17	482	no information	no information	surgery + 5-FU, surgery +5-FU + TAC	no information	87%	The combination of surgery with 5-FU reduced recurrence by 32%, and the best result was achieved when TAC was added.
Recurrence and Complications of Peri-operative Steroid Injection of Keloids: A Systematic Review and Meta-analysis	Yihan Zhang [25]	2024	systematic review	26	1663	no information	no information	triamcinolone + surgery	2.6% to 36%	up to 100%	Adjunctive therapies significantly reduce recurrence compared to surgery alone. recurrence rate was significantly lower with post-operative injection compared to intra-operative injection and pre-operative injection. A significant difference between intra-operative and pre-operative injection was not found. In conclusion, post-operative steroid injection after surgical excision results in lower keloid recurrence compared to pre- and intra-operative injection.
Could 5-Fluorouracil or Triamcinolone Be an Effective Treatment Option for Keloid After Surgical Excision? A Meta-Analysis	Jin Yong Shina [26]	2015	metanalysis	5	301	8–62	earlobe, chest, trunk, abdomen, face, scalp	surgery +5-FU, surgery + TAC	no information	no information	5-FU is effective as an adjuvant therapy; triamcinolone is not clearly better than surgery alone
Earlobe keloids: a pilot study of the efficacy of keloidectomy with core fillet flap and adjuvant intralesional corticosteroids	Ibrahim K Al Aradi [27]	2013	pilot study	no information	15	14–42 years old	earlobe	triamcinolone	earlobe keloids recur in 60% of patients treated using the standard excision	no information	Lower recurrence rates were achieved with surgery combined with steroid injections.
Imiquimod to prevent keloid recurrence postexcision: A systematic review and meta-analysis	Tanja Klotz [28]	2019	systematic review	7	77	>12	earlobe, chest, back, shoulder, neck	imiqumoid 5% cream	no data	no data	no renovation
A new uniform protocol of combined corticosteroid injections and ointment application reduces recurrence rates after surgical keloid/ hypertrophic scar excision.	Hayashi [29]	2012	interventional case series	-	24	11–79	suprapubic region, anterior chest, auricle excluding the earlobe, perineum, scapular region	corticosteroid injection combined with self-administered steroid ointment application	the recurrence rate when we previously used intralesional TA alone was 43% for three TA injections (3 of 7 cases), 33% for four injections (2 of 6 cases), 25% for five injections (2 of 8 cases), and 15% for six or more injections (2 of 13 cases), although we observed significantly more side effects requiring therapy discontinuation when we administered six or more intralesional TA injections.” “When using this [new] method, the recurrence rate was 14.3% to 16.7%	no information	Better outcomes with surgery + combined corticoid injections and ointment application.

**Table 2:** Summary of Treatment Methods, Timing of Triamcinolone Injection, and Recurrence Rates.

Title	Author et al.	Year of Publication	Days from Surgery to Triamcinolone Administration	Method I	Method I Patients, n	Timing–time from Surgery to Initiation of Method I (Days)	Recurrence Rate for Method I (%)	Method II	Method II Patients, n	Timing–Time from Surgery to Initiation of Method II (days)	Recurrence Rate for Method II (%)	Method III	Method III Patients, n	Timing–time from Surgery to Initiation of Method III (days)	Recurrence Rate for Method III (%)	Method IV	Method IV Patients, n	Timing–Time from Surgery to Initiation of Method IV (Days)	Recurrence Rate for Method IV (%)
Keloids and hypertrophic scars in individuals with darker Fitzpatrick skin types: a systematic review of treatment efficacy and quality of life outcomes	D. Reid [9]	2025	no information	radiation therapy + surgery	197	no information	2.8–83%	surgery + brachytherapy	43	no information	3.10%	surgery + triamcynolone	73	no information	0–33%	5-fluorouracil	4	no information	0%
Post-keloidectomy irradiation using high-dose-rate superficial brachytherapy	Shigehiko Kuribayash [10]	2011	no information	brachytherapy	21	no information	9.7	-	-	-	-	-	-	-	-	-	-	-	-
What Do We Know About Treating Recalcitrant Auricular Keloids? A Systematic Review and Meta-Analysis	Luke R. R. Zawadiuk [11]	2022	pre-operatively and postoperatively, intraoperatively, post-operatively in 1-month intervals or as 1 postoperative injection.	brachytherapy	no information	no information	10.5% ( primary ) i 15% (recalcitrant)	compression therapy	no information	no information	14%	external beam radiation therapy	no information	pre- and post-surgery.; 8 or 24 h after	17%	-	-	-	-
Surgical Excision with Adjuvant Irradiation for Treatment of Keloid Scars: A Systematic Review	Michiel CE van Leeuwen [12]	2015	no information	external radiation	98	-	<7–24 h 16.8–28.4%	HDR Brachytherapy	60	<7–24 h	10–10.7%	LDR brachytherapy	140	<7–24 h	19.4–22.3%	-	-	-	-
Combined surgical excision and radiation therapy for keloid treatment	Sadanori Akita [13]	2007	no information	surgery + radiotherapy	32	fourth day after surgery (the duration of electron beam radiation was 8.5 days (minimum 13 days; maximum 48 days))	21.1%	-	-	-	-	-	-	-	-	-	-	-	-
Postoperative radiation protocol for keloids and hypertrophic scars: statistical analysis of 370 sites followed for over 18 months	Rei Ogawa [14]	2007	no information	surgery + radiotherapy	218	third day after surgery	29.3%	-	-	-	-	-	-	-	-	-	-	-	-
				surgery + radiotherapy	109	fourth day after surgery	14%												
Wound Coverage, Adjuvant Treatments, and Surgical Outcomes for Major Keloid Scars: A Systematic Review and Meta-Analysis	David Cardenas [15]	2024	no information	surgery + post-excisional radiotherapy	457	radiotherapy treatment is sometimes deferred until ∼10 to 14 days after surgery, most effective when administered on the day of surgical excision and no later than 48 h postoperatively	23%	surgery + postoperative steroid and hyaluronidase injections	no information	every 14 days to every 90 days	no information	coverage techniques, including skin grafts, perforator flaps, closure with secondary intention, and skin substitute placement	no information	no information	no information	continuous pressure therapy	no information	no information	no information
Analysis of the surgical treatments of 63 keloids on the cartilaginous part of the auricle: effectiveness of the core excision method	Rei Ogawa [16]	2015	no information	radiotherapy	57	1,2,3 days after surgery	4.8%	-	-	-	-	-	-	-	-	-	-	-	-
Optimizing Radiotherapy for Keloids: A Meta-Analysis Systematic Review Comparing Recurrence Rates Between Different Radiation Modalities	Peter Mankowski [17]	2017	no information	radiotherapy	no information but of the 98 treatment groups, 71 groups were treated by surgical excision first followed by radiotherapy (postexcisional radiotherapy)	no information	22%	-	-	-	-	-	-	-	-	-	-	-	-
Chest keloids: effect of surgical excision and adjuvant radiotherapy on recurrence, a systematic review and meta-analysis	Oliver J. Miles [18]	2021	no information	radiation	400	no information	22%	-	-	-	-	-	-	-	-	-	-	-	-
Effectiveness of Core Excision Technique for Keloids: A Systematic Review	Xiaoye Ran [19]	2024	no information	surgery + radiotherapy	no information	no information	0–14.1%,	surgery + steroid injections	no information	no information	0–18.8%	-	-	-	-	-	-	-	-
A Comparison of the Effectiveness of Triamcinolone and Radiation Therapy for Ear Keloids after Surgical Excision: A Systematic Review and Meta-Analysis	Jin Yong Shin [20]	2016	14–90 days	surgery + triamcynolon (preoperative, intraoperative, postoperative)	no information	7–90 days	15.4 (intraoperative + postoperative 20.8%, postoperative only 15.2%, preoperative + intraoperative + postoperative 5.3%)	surgery + radiotherapy	no information	3 h–7 days	14%	-	-	-	-	-	-	-	-
The Efficacy of Surgical Excision Plus Adjuvant Multimodal Therapies in the Treatment of Keloids: A Systematic Review and Meta-Analysis	Morgan M Ellis [21]	2020	different protocols, no average	surgery + radiotherapy	dual therapy various combo 5243 keloids, no information about number of patients	often within 24–72h	18.70%	surgery + TAC	dual therapy various combo 5243 keloids, no information about number of patients	no information	21.70%	surgery + radiotherapy + 3rd therapy: HBU, presure, PRP, 5-FU	no information about number of patients, total number of keloids from triple therapy is 259	no information	11.20%	surgery + TAC + 3rd: cryo, 5-fu, silicone, pressure, PRP	no information about number of patients, total number of keloids from triple therapy is 259	no information	13.8%
Effect of the biologically effective dose of electron beam radiation therapy on recurrence rate after keloid excision: A meta-analysis	Na-Hyun Hwang [22]	2022	no information	surgery + radiotherapy	no informationrmtion about patients but 3128 keloids	0–72 h	body 16%, ear 11%	-	-	-	-	-	-	-	-	-	-	-	-
Compression Therapy for Keloid Scars: A Systematic Review and Meta-analysis	Sadia M Tahir [23]	2024	not applicable	surgery + pressure earing	2026	no information	10.66%	surgery + silicone gel	179	no information	12.86%	surgery + pressure earing + silicone gel	76	no information	9.09%	-	-	-	-
Intralesional 5-fluorouracil in keloid treatment: a systematic review	Eveline Bijlard [24]	2015	in day 7, 14, 28, 56, 84 after surgery	surgery + 5-FU	171	most often just after surgery	19%, 4%	surgery + 5-FU + TAC	24	in day 0, 28, 56	no information	-	-	-	-	-	-	-	-
Recurrence and Complications of Peri-operative Steroid Injection of Keloids: A Systematic Review and Meta-analysis	Yihan Zhang [25]	2024	postoperative 7–21	triamcinolone + surgery	684	postoperative 7–21	preoperation 9.9% intraoperation 12.7% postoperation 0.9% overall 6.6%	-	-	-	-	-	-	-	-	-	-	-	-
Could 5-Fluorouracil or Triamcinolone Be an Effective Treatment Option for Keloid After Surgical Excision? A Meta-Analysis	Jin Yong Shina [26]	2015	weekly or monthly incjections so first given 7 or 30 days after surgery	surgeru + 5-FU	107	mostly day 0 (just after surgery)	no information	surgery + TAC	254	weekly or monthly incjections so first given 7 or 30 days after surgery	no information	-	-	-	-	-	-	-	-
Earlobe keloids: a pilot study of the efficacy of keloidectomy with core fillet flap and adjuvant intralesional corticosteroids	Ibrahim K Al Aradi [27]	2013	intraoperative injection	surgery + triamcinolone	15	intraoperative injection, then injection every 1 month (a mean number of postoperative intralesional corticosteroid injections of 6.8 (range 1–13))	9.5%	-	-	-	-	-	-	-	-	-	-	-	-
Imiquimod to prevent keloid recurrence postexcision: A systematic review and meta-analysis	Tanja Klotz [28]	2019	not applicable	surgery + imiquimod 5% cream	77	varies from immediately to 7 days	39%: earlobe 5.4%, other locations 76.8%	-	-	-	-	-	-	-	-	-	-	-	-
A new uniform protocol of combined corticosteroid injections and ointment application reduces recurrence rates after surgical keloid/ hypertrophic scar excision.	Hayashi [29]	2012	one injection at the time of suture removal and injections every 2 weeks thereafter for a total of five injections (injection included 1 mL triamcinolone and 1 mL procaine hydrochloride)	corticosteroid injection combined with self-administered steroid ointment application	24	7	14.3% for keloids, 16.7% hypetrophic scars	-	-	-	-	-	-	-	-	-	-	-	-

### 3.3. Surgical Methods

Most studies did not specify the surgical techniques for keloid removal. One study used tangential/shaving excision. Three studies described the use of core excision as the primary surgical approach.

## 4. Discussion

The pathogenesis of keloid formation is primarily driven by excessive fibroblast proliferation, mediated by a complex interplay of pro-inflammatory and anti-inflammatory cytokines, chemokines, and growth factors, resulting in abnormal collagen synthesis and deposition [30]. Additional contributing factors include immunological, genetic, endocrine, and mechanical influences such as skin tension and trauma [31].

Despite extensive investigation, no definitive gold-standard treatment has been established that reliably eliminates keloids without recurrence [32]. Surgical treatment continues to be regarded as a promising modality, as it provides immediate relief from discomfort such as pain and pruritus; however, when used as monotherapy recurrence rates range from 45% to 100%, underscoring the necessity of adjuvant treatment strategies [1].

This systematic review synthesizes the evidence supporting the effectiveness of combining surgical excision with adjuvant physical therapy and pharmacology. Although differences in lesion severity, anatomical location, and treatment protocols complicate direct comparisons, the results consistently indicate that surgery is an effective primary intervention [33].

One significant limitation of the study is the lack of detailed description of the excision techniques used, leading to heterogeneous and inconsistent results and hindering the selection of the optimal treatment modality.

The follow-up period was reported in 17 studies and ranged from 3–6 months to 16 years. The results are too heterogeneous to allow for evaluation. This undoubtedly highlights the opportunity to conduct a prospective study that would enable a reliable assessment of the efficacy of a specific adjunctive treatment for keloid management.

Radiotherapy is considered a valuable therapeutic option for keloids that do not respond adequately to conventional treatments [34]. By inhibiting fibroblast proliferation, it disrupts the excessive wound-healing response and helps prevent recurrent lesion formation [33]. However, optimal timing of postoperative irradiation remains controversial [35]. In the present analysis, radiotherapy was most frequently initiated within the first 72 h after surgery, producing recurrence rates between 14% and 29.3%. Renu Sah et al. demonstrated that irradiation delivered on the first postoperative day ideally within two hours produced the most favourable outcomes, with recurrence rates of 5–7%. In contrast, delaying treatment beyond six hours, however, led to significantly poorer outcomes [36]. Conversely, Chin-Ling Hsieh and colleagues found no significant differences between irradiation performed within 24 h and later postoperative administration [37]. These findings collectively indicate that considerable uncertainty remains regarding the optimal timing of radiotherapy, and this topic continues to attract substantial research interest [35].

The two principal radiotherapy modalities employed in keloid management are brachytherapy and external beam radiotherapy (EBRT) [38]. Brachytherapy delivers gamma radiation directly to the targeted tissue while minimizing exposure to surrounding structures. This method is particularly useful for irregular anatomical regions that would otherwise necessitate multiple radiation fields [39]. Evidence summarized in our review indicates that postoperative brachytherapy is associated with comparatively lower recurrence rates (9.7–15%) than EBRT (14–29.3%) [40].

In contrast, EBRT typically requires higher radiation doses to achieve equivalent therapeutic outcomes. This modality remains well suited for treating more superficial lesions [41].

Compression therapy following surgical excision demonstrated recurrence rates of 10.66% and 14% in the studies included in our analysis. Its proposed mechanism involves inducing localized hypoxia, which promotes fibroblast apoptosis, while increased collagenase activity contributes to stabilization of the scar tissue [23]. This approach appears particularly effective for auricular keloids when combined with surgery, achieving recurrence rates as low as 6.7–10.6% [42].

Pharmacological therapies play a crucial role in the management of keloids. In this review, we summarize evidence regarding the use of triamcinolone acetonide (TAC), 5-fluorouracil (5FU), and 5% imiquimod.

TAC is widely regarded as the primary non-surgical treatment option for keloids [43]. Its therapeutic effect is believed to stem from the suppression of inflammatory signalling pathways as well as the inhibition of fibroblast activity and collagen synthesis [42]. Across the studies included in our review, TAC was administered intralesional according to various postoperative schedules, most commonly at intervals of 7–30 days, although some protocols extended the interval to 14–90 days or incorporated intraoperative administration. The lowest recurrence rate observed was 6.6%. In the study by Young-Jun Choi et al., combined therapy achieved a recurrence rate of 5% during one year of follow-up [44]. TAC is frequently combined with 5-FU, as this regimen has demonstrated superior efficacy and safety compared with monotherapy [45,46].

5-FU acts as an antimetabolite that inhibits fibroblast proliferation by disrupting RNA synthesis and reducing type I collagen gene expression [45]. In most of the studies assessed in our review, the use of 5-FU as an adjuvant treatment resulted in no reported recurrences, although the follow-up duration varied considerably. One study documented a reduction in recurrence rates from 87% to 32% when compared with surgery alone. Findings from Steven P. Davison et al. further demonstrated that combining surgery with TAC and 5-FU produced excellent outcomes a mean 92% reduction in lesion size-compared with 81% when pharmacologic therapy was used without surgery [47].

Imiquimod, a potent immune response modifier, is typically applied topically to the excision site for 6–8 weeks. A recent review reported a recurrence rate of 24.7% at six months of follow-up [42], whereas our analysis identified a recurrence rate of 14.3%.

### Limitations

Unfortunately there are some limitations in our work. The studies included in our review did not categorise patients as individual units, but treated them as a group. Nor did they provide a breakdown by race, information on the comorbidities of the patients included, or whether they were taking long-term medication. Information on specific drug manufacturers was inconsistently reported across studies and was therefore not systematically analyzed.

## 5. Conclusions

Surgical excision combined with adjuvant therapy remains the most effective strategy for keloid management. Evidence from this systematic review indicates that multimodal approaches significantly reduce recurrence rates compared with surgery alone. Among physical modalities, postoperative brachytherapy appears to provide the lowest recurrence rates, while EBRT remains a viable option for selected superficial lesions. Pharmacological adjuvants, particularly triamcinolone acetonide combined with 5-fluorouracil, demonstrate superior efficacy compared with monotherapy. However, substantial heterogeneity in treatment protocols, timing, and lesion characteristics limits direct comparison across studies. Further high-quality, standardized clinical trials are required to establish optimal treatment algorithms and define evidence-based guidelines for keloid management.

## Data Availability

No new data were created or analyzed in this study.

## Supplementary Materials

The following supporting information can be downloaded at: https://www.mdpi.com/article/10.3390/medicina62050916/s1, PRISMA 2020 Checklist [8]; Table S1: Summary of Included Studies.

## References

[1] Fu S., Duan L., Zhong Y., Zeng Y. (2024). Comparison of surgical excision followed by adjuvant radiotherapy and laser combined with steroids for the treatment of keloids: A systematic review and meta-analysis. Int. Wound J..

[2] Zhao J., Zhai X., Xu Z., Zhou S., Gu L., Chen L., Zhou B., Hua H. (2024). Novel needle-type electrocoagulation and combination pharmacotherapy: Basic and clinical studies on efficacy and safety in treating keloids. J. Cosmet. Dermatol..

[3] Limandjaja G.C., Niessen F.B., Scheper R.J., Gibbs S. (2021). Hypertrophic scars and keloids: Overview of the evidence and practical guide for differentiating between these abnormal scars. Exp. Dermatol..

[4] Gauglitz G.G., Korting H.C., Pavicic T., Ruzicka T., Jeschke M.G. (2011). Hypertrophic Scarring and Keloids: Pathomechanisms and Current and Emerging Treatment Strategies. Mol. Med..

[5] Kim H.J., Kim Y.H. (2024). Comprehensive Insights into Keloid Pathogenesis and Advanced Therapeutic Strategies. Int. J. Mol. Sci..

[6] Jagdeo J., Kerby E., Glass D.A. (2021). Keloids. JAMA Dermatol..

[7] Qi W., Xiao X., Tong J., Guo N. (2023). Progress in the clinical treatment of keloids. Front. Med..

[8] Page M.J., McKenzie J.E., Bossuyt P.M., Boutron I., Hoffmann T.C., Mulrow C.D., Shamseer L., Tetzlaff J.M., Akl E.A., Brennan S.E. (2021). The PRISMA 2020 statement: An updated guideline for reporting systematic reviews. BMJ.

[9] Reid D., Malak S., Khadka M., Hanna R., Pharr T., Wyant W.A., Albers S. (2025). Keloids and hypertrophic scars in individuals with darker Fitzpatrick skin types: A systematic review of treatment efficacy and quality of life outcomes. Arch. Dermatol. Res..

[10] Kuribayashi S., Miyashita T., Ozawa Y., Iwano M., Ogawa R., Akaishi S., Dohi T., Hyakusoku H., Kumita S. (2011). Post-keloidectomy irradiation using high-dose-rate superficial brachytherapy. J. Radiat. Res..

[11] Zawadiuk L.R.R., Van Slyke A.C., Bone J., Redfern B., Carr N.J., Arneja J.S. (2022). What Do We Know About Treating Recalcitrant Auricular Keloids? A Systematic Review and Meta-Analysis. Plast. Surg..

[12] Van Leeuwen M.C.E., Stokmans S.C., Bulstra A.E.J., Meijer O.W.M., Heymans M.W., Ket J.C.F., Ritt M.J.P.F., van Leeuwen P.A.M., Niessen F.B. (2015). Surgical excision with adjuvant irradiation for treatment of keloid scars: A systematic review. Plast. Reconstr. Surg.–Glob. Open.

[13] Akita S., Akino K., Yakabe A., Imaizumi T., Tanaka K., Anraku K., Yano H., Hirano A. (2007). Combined surgical excision and radiation therapy for keloid treatment. J. Craniofacial Surg..

[14] Ogawa R., Miyashita T., Hyakusoku H., Akaishi S., Kuribayashi S., Tateno A. (2007). Postoperative radiation protocol for keloids and hypertrophic scars: Statistical analysis of 370 sites followed for over 18 months. Ann. Plast. Surg..

[15] Cardenas D., Cinclair T., Dogaroiu A., Hinson C., Sink M., Bruce B., Odobescu A., Sammer D., Zhang A.Y. (2024). Wound Coverage, Adjuvant Treatments, and Surgical Outcomes for Major Keloid Scars: A Systematic Review and Meta-Analysis. Aesthetic Surg. J. Open Forum.

[16] Ogawa R., Akaishi S., Dohi T., Kuribayashi S., Miyashita T., Hyakusoku H. (2015). Analysis of the Surgical Treatments of 63 Keloids on the Cartilaginous Part of the Auricle. Plast. Reconstr. Surg..

[17] Mankowski P., Kanevsky J., Tomlinson J., Dyachenko A., Luc M. (2017). Optimizing Radiotherapy for Keloids: A Meta-Analysis Systematic Review Comparing Recurrence Rates between Different Radiation Modalities. Ann. Plast. Surg..

[18] Miles O.J., Zhou J., Paleri S., Fua T., Ramakrishnan A. (2021). Chest keloids: Effect of surgical excision and adjuvant radiotherapy on recurrence, a systematic review and meta-analysis. ANZ J. Surg..

[19] Ran X., Guo X., Liu Y., Zhu S., Li S., Chen Z., Han T., Jin S., Zhou M., Zang M. (2025). Effectiveness of Core Excision Technique for Keloids: A Systematic Review. Plast. Reconstr. Surg..

[20] Shin J.Y., Lee J.W., Roh S.G., Lee N.H., Yang K.M. (2016). A Comparison of the Effectiveness of Triamcinolone and Radiation Therapy for Ear Keloids after Surgical Excision: A Systematic Review and Meta-Analysis. Plast. Reconstr. Surg..

[21] Ellis M.M., Jones L.R., Siddiqui F., Sunkara P.R., Ozog D.M. (2020). The efficacy of surgical excision plus adjuvant multimodal therapies in the treatment of keloids: A systematic review and metaanalysis. Dermatol. Surg..

[22] Hwang N.H., Chang J.H., Lee N.K., Yang K.S. (2022). Effect of the biologically effective dose of electron beam radiation therapy on recurrence rate after keloid excision: A meta-analysis. Radiother. Oncol..

[23] Tahir S.M., Ihebom D., Simman R. (2024). Compression Therapy for Keloid Scars: A Systematic Review and Meta-analysis. Plast. Reconstr. Surg. Glob. Open.

[24] Bijlard E., Steltenpool S., Niessen F.B. (2015). Intralesional 5-fluorouracil in keloid treatment: A systematic review. Acta Derm. Venereol..

[25] Zhang Y., Wu M., Liu D., Panayi A.C., Xu X., Luo L., Feng J., Ou Y., Lin T., Cui Y. (2024). Recurrence and Complications of Peri-operative Steroid Injection of Keloids: A Systematic Review and Meta-analysis. Aesthetic Plast. Surg..

[26] Shin J.Y., Kim J.S. (2016). Could 5-Fluorouracil or Triamcinolone Be an Effective Treatment Option for Keloid after Surgical Excision? A Meta-Analysis. J. Oral Maxillofac. Surg..

[27] Al Aradi I.K., Alawadhi S.A., Alkhawaja F.A. (2013). Earlobe keloids: A pilot study of the efficacy of keloidectomy with core fillet flap and adjuvant intralesional corticosteroids. Dermatol. Surg..

[28] Klotz T., Munn Z., Aromataris E.C., Greenwood J.E. (2020). Imiquimod to prevent keloid recurrence postexcision: A systematic review and meta-analysis. Wound Repair Regen..

[29] Hayashi T., Furukawa H., Oyama A., Funayama E., Saito A., Murao N., Yamamoto Y. (2012). A New Uniform Protocol of Combined Corticosteroid Injections and Ointment Application Reduces Recurrence Rates After Surgical Keloid/Hypertrophic Scar Excision. Dermatol. Surg..

[30] Liu M. (2025). Cytokines, chemokines and growth factors involved in keloids pathogenesis. An. Bras. Dermatol..

[31] Xia G., Dohi T., Abdelhakim M., Tosa M., Ogawa R. (2024). The effects of systemic diseases, genetic disorders and lifestyle on keloids. Int. Wound J..

[32] Ojeh N., Bharatha A., Gaur U., Forde A.L. (2020). Keloids: Current and emerging therapies. Scars Burn. Heal..

[33] Ekstein S.F., Wyles S.P., Moran S.L., Meves A. (2021). Keloids: A review of therapeutic management. Int. J. Dermatol..

[34] Walsh L.A., Wu E., Pontes D., Kwan K.R., Poondru S., Miller C.H., Kundu R.V. (2023). Keloid treatments: An evidence-based systematic review of recent advances. Syst. Rev..

[35] Dong W., Qiu B., Fan F. (2022). Adjuvant Radiotherapy for Keloids. Aesthetic Plast. Surg..

[36] Peng Q., Lu Y., Huang R., Chen R. (2025). Should We Do Postoperative Radiotherapy After Keloid Excision as Soon as Possible? A Systematic Review and Meta-Analysis. Aesthetic Plast. Surg..

[37] Hsieh C.L., Chi K.Y., Lin W.Y., Lee L.T.J. (2021). Timing of Adjuvant Radiotherapy After Keloid Excision: A Systematic Review and Meta-Analysis. Dermatol. Surg..

[38] Liu E.K., Cohen R.F., Chiu E.S. (2022). Radiation therapy modalities for keloid management: A critical review. J. Plast. Reconstr. Aesthetic Surg..

[39] Bryant C., Mendenhall W.M. (2017). Radiation Therapy. Juvenile Angiofibroma.

[40] Frech F.S., Hernandez L., Urbonas R., Zaken G.A., Dreyfuss I., Nouri K. (2023). Hypertrophic Scars and Keloids: Advances in Treatment and Review of Established Therapies. Am. J. Clin. Dermatol..

[41] Goutos I., Ogawa R. (2017). Brachytherapy in the adjuvant management of keloid scars: Literature review. Scars Burn. Heal..

[42] Thornton N.J., Garcia B.A., Hoyer P., Wilkerson M.G. (2021). Keloid Scars: An Updated Review of Combination Therapies. Cureus.

[43] Morelli Coppola M., Salzillo R., Segreto F., Persichetti P. (2018). Triamcinolone acetonide intralesional injection for the treatment of keloid scars: Patient selection and perspectives. Clin. Cosmet. Investig. Dermatol..

[44] Choi Y., Lee Y.H., Lee H.J., Lee G., Kim W. (2020). Auricular keloid management in Asian skin: Clinical outcome of intralesional excision and postoperative triamcinolone acetonide intralesional injection. J. Cosmet. Dermatol..

[45] Srivastava S., Patil A.N., Prakash C., Kumari H. (2017). Comparison of Intralesional Triamcinolone Acetonide, 5-Fluorouracil, and Their Combination for the Treatment of Keloids. Adv. Wound Care.

[46] Wu W., Zhao Y., Chen Y., Zhong A. (2023). Comparing the Efficacy of Multiple Drugs Injection for the Treatment of Hypertrophic Scars and Keloid: A Network Meta-Analysis. Aesthetic Plast. Surg..

[47] Davison S.P., Dayan J.H., Clemens M.W., Sonni S., Wang A., Crane A. (2009). Efficacy of Intralesional 5Fluorouracil and Triamcinolone in the Treatment of Keloids. Aesthetic Surg. J..

